# Senescence, regulators of alternative splicing and effects of trametinib treatment in progeroid syndromes

**DOI:** 10.1007/s11357-023-00933-z

**Published:** 2023-09-26

**Authors:** Laura R. Bramwell, Lorna W. Harries

**Affiliations:** RNA-Mediated Mechanisms of Disease Group, Department of Clinical and Biomedical Sciences (Medical School), Faculty of Health and Life Sciences, University of Exeter, Exeter, UK

**Keywords:** Progeria, Human, Ageing, Dermal fibroblast, Senescence, Splicing factor

## Abstract

Progeroid syndromes such as Hutchinson Gilford Progeroid syndrome (HGPS), Werner syndrome (WS) and Cockayne syndrome (CS), result in severely reduced lifespans and premature ageing. Normal senescent cells show splicing factor dysregulation, which has not yet been investigated in syndromic senescent cells. We sought to investigate the senescence characteristics and splicing factor expression profiles of progeroid dermal fibroblasts. Natural cellular senescence can be reversed by application of the senomorphic drug, trametinib, so we also investigated its ability to reverse senescence characteristics in syndromic cells. We found that progeroid cultures had a higher senescence burden, but did not always have differences in levels of proliferation, DNA damage repair and apoptosis. Splicing factor gene expression appeared dysregulated across the three syndromes. 10 µM trametinib reduced senescent cell load and affected other aspects of the senescence phenotype (including splicing factor expression) in HGPS and Cockayne syndromes. Werner syndrome cells did not demonstrate changes in in senescence following treatment. Splicing factor dysregulation in progeroid cells provides further evidence to support this mechanism as a hallmark of cellular ageing and highlights the use of progeroid syndrome cells in the research of ageing and age-related disease. This study suggests that senomorphic drugs such as trametinib could be a useful adjunct to therapy for progeroid diseases.

## Introduction

The human ageing process is complex and occurs over decades, but insight can be gained by studying the rare monogenic conditions that result in vastly accelerated rates of ageing [1]. These conditions often result from errors in the gene regulatory or DNA damage response machinery and are collectively termed progeroid syndromes. Despite often being extremely rare, the syndromes are life-changing and can be fatal [2, 3]. Some of the most studied progeroid syndromes are Hutchinson-Gilford Progeria syndrome (HGPS), Werner syndrome (WS) and Cockayne syndrome (CS). These are rare disorders affecting an estimated 1 in 18 million live births for HGPS and 1 in 200,000 for both WS and CS [2, 4–6].

HPGS is caused by mutations in the Lamin A (*LMNA*) gene that result in a truncated Lamin A protein termed progerin [7]. The genetics of WS is also well defined, with the syndrome caused by mutations in the Werner (*WRN*) gene which encodes the DNA helicase, Werner protein [5]. CS is more genetically and phenotypically diverse, with several subtypes across a spectrum of distinguishing phenotypic features; CS type I (moderate disease/classical phenotype), type II (severe or early-onset), type III (mild), photosensitivity only (adult onset) and cerebraloculofacioskeletal syndrome (COFS; severe, foetal onset) [6, 8]. No correlation has yet been found linking the phenotypic subtypes with two known genetic mechanisms of Cockayne syndrome: CS type A and B. The two genetic causes correspond to mutations in excision repair genes *ERCC8* (Cockayne syndrome A) or *ERCC6* (Cockayne syndrome B) [6, 8].

All of the three syndromes share common features of accelerated ageing and significantly higher risks of age-related diseases such as cardiovascular disease, cancer, diabetes and chronic kidney disease [3, 9, 10]. However, the severity of phenotypes between HGPS, WS and CS demonstrate differences. The defects in *LMNA*, *WRN* and *ERCC6/8* seen in HGPS, WS and CS, respectively, all cause major instability of the nuclear envelope and large scale unrepaired genomic damage, resulting in major curtailment of average lifespan to 14.5 years for HGPS and 54 years for WS [3, 5, 10, 11]. The severity of CS affects the observed mean lifespan across the different phenotypic subtypes. People with CS type I have a mean lifespan of 16.1 years, those with CS type II have a mean lifespan of 5 years, and those with type III have a mean lifespan of 30.3 years [8]. In this study, we examine the mildest subtype, CS type III, which enables a contrast with two more severe syndromes: HGPS and WS. The CS type III subtype is generally milder with patients given an expected lifespan of 10—30 years and milder symptoms of accelerated ageing, although they remain abnormally sensitive to UV-induced DNA damage [6, 8]. Despite their monogenic cause, the molecular features of ageing observed in progeroid syndromes are similar to those seen during normal ageing. Assessment of potential therapeutics to slow rates of ageing (senotherapeutics) in progeroid syndromes may indicate interventions with efficacy against common, chronic ageing diseases in the wider population.

There are several theories as to how and why we age. Evidence is mounting that the physiological and functional changes that occur during the ageing process arise from the gradual failure of a series of basic health maintenance mechanisms. These mechanisms are termed the hallmarks of ageing, which are also major drivers of age-related disease [12, 13]. Hallmarks include genomic instability, epigenetic alterations, mitochondrial dysfunction, altered intercellular communication, deregulated proteostasis, deregulated nutrient sensing, telomere attrition, stem cell exhaustion, dysregulated regulation of alternative splicing, compromised autophagy, altered mechanical properties, disturbances to the microbiome, inflammation and cellular senescence. They are present in normal ageing in multiple species, and underpin many of the common chronic diseases of human ageing [12]. Importantly, the hallmarks of ageing represent promising avenues for therapeutic targeting of the diseases of ageing. This is exemplified by observations that targeted depletion of senescent cells leads to increases in multiple healthspan and lifespan-related parameters in animal ageing/progeria models [14–16]. In smaller studies, targeted reduction of senescence can affect the phenotype of idiopathic pulmonary fibrosis and diabetic kidney disease in humans [17, 18].

More recently, dysregulation of the expression of splicing regulatory factors has been proposed as a new hallmark of ageing, since it is known to be associated with normal human ageing [13, 19–21]. mRNA splicing is a carefully controlled mechanism by which our genes can produce many different mRNA transcripts [22]. It is controlled by a portfolio of splicing factors which are themselves regulated by alternative splicing; splicing factors are proteins which act on the gene to influence a change in the splice site of a pre-mRNA transcript. In line with its recent designation as a novel hallmark, dysregulation of splicing factor expression occurs during normal ageing in multiple species, its experimental aggravation induces cellular senescence and ageing phenotypes, and its experimental amelioration alters aspects of cellular and organismal ageing in human cells and in other species [20, 23–31]. Dysregulated splicing factor expression arises from unresolved and constitutive signalling through ERK and AKT pathways, culminating in altered activity or expression of the *FOXO1* and *ETV6* genes [32]. The fruit fly homologues of these genes (*Foxo* and *Aop*) have also previously been demonstrated to be the genetic effectors of RAS/MEK/ERK and PI3K/AKT signalling in relation to lifespan in *Drosophila melanogaster* [33].

New research into senotherapeutic compounds that modulate senescence-related pathways may be an important avenue for future therapies for progeroid diseases. Compounds with senomorphic (reversal of senescence) or senolytic (lysis of senescent cells) properties may have effects on the premature ageing phenotype seen in the progeroid syndromes. Drugs with senomorphic properties like resveratrol and rapamycin affect several senescence-associated pathways, but can have pleiotropic effects [34]. Some compounds that inhibit p38/MAPK have been suggested to aid in the treatment of WS, however the potential therapeutic effects of inhibiting other senescence pathways have not yet been investigated for WS [35–37]. Only lonafarnib, a farnesyltransferase inhibitor, is approved for HGPS, but several senotherapeutic compounds like rapamycin have been identified as having potential in in vitro research [38–42]. The action of the farnesyltransferase is able to improve the persistent farnesylation of the aberrant Lamin A protein caused by HGPS [38].

Senotherapeutic compounds can be used to target specific aspects of the senescence phenotype. For example, trametinib is a drug which specifically inhibits both isoforms of MEK (MEK1 and MEK2) and it has been approved by the FDA for the treatment of metastatic melanoma [43–45]. The effects of trametinib on splicing factor expression have been well characterised in previous work, so we know that with the application of low dose (1—10 µM) trametinib to senescent primary human dermal fibroblasts, we are able to restore splicing factor expression and bring about a reversal of several aspects of the senescent cell phenotype [32]. Drugs like trametinib may help reduce some of the senescent phenotype of progeroid diseases.

Here, we aimed firstly to determine whether disrupted splicing factor expression is a feature of the accelerated ageing phenotypes seen in progeroid syndromes, as it is for normal ageing [23]. Secondly, should splicing factor profiles be disrupted in progeroid cells, we aimed to determine whether trametinib was capable of restoring correct expression of splicing factors and attenuating senescence phenotypes in progeroid cells, as it does in wild-type cells [32]. This study could help elucidate novel mechanisms of senescence and identify a future point of therapeutic intervention for progeroid diseases.

## Materials & Methods

### Human primary cells

All cells used in this study were commercially derived, with ethical permission granted at source. Normal human dermal fibroblasts (nHDF) were purchased from Promocell, Heidelberg (catalogue number C-12302, lot number 445Z026.3). The donor was male, Caucasian and 36 years old at donation. Cells were taken from the abdomen. At the time of these experiments, nHDF cells had cumulated population doublings (cPDL) of 29.44. All three progeroid syndrome cell lines were human primary dermal fibroblasts purchased from the Coriell Institute (Camden, New Jersey, United States). Cells from an HGPS donor (catalogue number AG06917) were from a 3-year-old Caucasian male and were taken from the patient’s arm. The cells have a normal karyotype (46, XY), but have a de novo single point mutation (2036 C > T) in the Lamin A (*LMNA*) gene. The patient displayed reduced subcutaneous tissue, thin skin, a thin beak-like nose characteristic of HGPS, thin underdeveloped nails, narrow clavicles, and growth retardation. HGPS cells had a cPDL of 6.36 for the characterisation of untreated cells, and 36.30 at the time of the trametinib treatment experiments.

Cells from a donor with WS (catalogue number AG05233) were from a 36-year-old male Asian patient. The karyotype of this patient is reported by the Coriell Institute as:

- 46,XY,t(1;9)(1qter > 1p32::9q22 > 9qter;9pter > 9q22::1q32 > 1qter),t(1;2;5)(1pter > 1q21::5q11.2 > 5qter;2pter > 2q13::1q21 > 1qter;5pter > 5q11.2::2q13 > 2qter),t(5;10)(5pter > 5q11.2::10p15 > 10pter;10qter > 10p15::5q11.2 > 5qter),inv(13)(pter > p21::q34 > q21::q34 > qter[43]/46,XY.

Data for the exact mutation in the *WRN* gene was not available. Mutations in the *WRN* gene typically cause truncation of the Werner protein [46]. The donor had a short stature, grey hair, skin hyperpigmentation, atrophic skin and subcutaneous tissue, hypogonadism, cataracts and diabetes. Cells were taken from the patient’s thigh. 40% of cells show random chromosomal abnormalities, but the remainder have a normal karyotype (46, XY). For both the characterisation of untreated cells and the trametinib treatment experiments, WS cells had a cPDL of 9.00.

Cells from a CS donor (catalogue number AG07076) were donated by an 11-year-old Caucasian female with CS-type A/type III. The patient had the least severe phenotypic type of CS: CS type III. They had a phenotype of dwarfism, mental retardation, cataracts, photophobia, retinopathy and optic atrophy. Being CS type A, the cells have a mutation in the *ERCC8* gene. This donor had a normal karyotype (46XX) but demonstrated compound heterozygosity for two mutations: a 649G-C transversion, and a G-to-T transversion. These mutations result in an ala205-to-pro (A205P) substitution, and a glu13-to-ter (E13X) substitution, respectively [47, 48]. CS cells had a cPDL of 11.32 for the characterisation of untreated cells, and 18.63 at the time of the trametinib treatment.

### Tissue culture

All cells were grown in animal component-free conditions. Cells were cultured in DMEM 1 g/l glucose + phenol red (31885023, Gibco™), 10% human serum (H3667, Sigma Aldrich) and 1% 10,000 U/ml penicillin—10,000 µg/ml streptomycin (15140122, Gibco™). We used TryPLE™ Express (12604013, Gibco™) to detach cells. A Hirschmann haemocytometer was used to perform cell counts, which together with the cPDL numbers given by Promocell and the Coriell Institute, enabled assessment of cPDL at the time of seeding for experiments. Cells were transferred to antibiotic-free media for 48 to 72 h before seeding. Cells were seeded at approximately 30,000 cells/well in 12-well plates for staining experiments. For harvesting RNA for RT-qPCR analysis, cells were seeded at approximately 50,000 cells/well in 6-well plates. Dosing for trametinib studies was taken from our previous work, where a 10 µM dose suspended in 10% DMSO (HY-10999, MedchemExpress; J66650.AD, Thermo Scientific Alfa Aesar) resulted in attenuation of splicing factor expression and rescue of aspects of the senescent cell phenotype in wild-type human primary dermal fibroblasts [28].

### Senescence-associated beta galactosidase (SAB) experiments and analysis

We used the Senescence Cells Histochemical Staining kit (CS0030, Merck) as per the manufacturer’s instructions to stain senescence-associated beta galactosidase (SAB). 24 h after staining, cells were imaged using Zeiss AxioCam ERC55 PrimoVert at 10 × magnification. Five images per biological replicate were later counted manually using ImageJ 1.47v software (US National Institute of Health, Bethesda, Maryland, USA) [49].

### Immunocytochemical staining experiments and analysis

Cells were grown on 13 mm coverslips in 12-well plates, washed in DPBS (14190136, Gibco™) before fixation with 4% paraformaldehyde and storage in DPBS. Before immunocytochemical staining, cells were washed in DPBS, and blocked using ADST [antibody diluent solution—triton: DPBS, 0.1 M L-Lysine (303341000, Thermo Scientific™), 1% w/v Human Serum Albumin Fraction V (12668-10GM, Sigma-Aldrich), Triton X-100 (A16046.AP, Thermo Scientific Alfa Aesar)] and 5% human serum (H3667, Sigma Aldrich) for 30 min. Cells were washed and primary antibodies at 2.5 µg/ml (suspended in ADST with 2% human serum) were applied overnight. After washing, secondary antibodies at 5 µg/ml and 4′,6-diamidino-2-phenylindole (DAPI, D1306, Invitrogen™) at 1 µg/ml (suspended in ADST with 2% human serum) were applied for 1 h, before mounting coverslips using Dako mounting medium (S302380-2, Agilent). Antibodies were sourced from Abcam: Rb anti-Ki67 (ab15580, ab16667), Ms anti-γH2AX (ab26350), Alexa Fluor ® 555 Goat pAb to Rb (ab150078, ab150086) and Alexa Fluor ® 488 Goat pAb to Ms (ab150117). Images were captured using the Leica DM4 B Upright Microsope at 10 × magnification and were counted manually using Leica Application Suite X 2019 3.7.1.21655v software (Leica Microsystems, Wetzlar, Germany).

### RNA extraction

Cells were washed twice in DPBS (14190136, Gibco™) and removed from the culture plate by cell scraping in TRI Reagent Solution (AM9738, Invitrogen™) supplemented with 10 mM MgCl_2_ (AM9530G, Invitrogen™). RNA extraction was carried out with phase separation using chloroform (C/4920/08, Fisher Chemical) and precipitation with an equivalent volume of 100% v/v isopropanol (BP2618-1, Fisher Bioreagents) and 1.2 µl of 15 mg/ml GlycoBlue™ Coprecipitant (AM9516, Invitrogen™) to aid the recovery of the pellet. After two 75% ethanol washes, the pellets were resuspended in 20 µl 1 × TE buffer, pH 8.0 (BP2473-500, Fisher Bioreagents). The quality and concentration of RNA were checked using the Thermo Scientific™ Nanodrop 8000 Spectrophotometer (Thermo Fisher Scientific, Waltham, Massachusetts, USA).

### Reverse transcription and pre-amplification

cDNA was produced by reverse transcription using the High-Capacity cDNA Reverse Transcription Kit (4368813, Applied Biosystems™) following the manufacturer’s instructions using an Applied Biosystems™ Veriti™ 96-Well Fast Thermal Cycler. Cycling conditions were: 25 °C for 10 min, 37 °C for 120 min and 85 °C for 5 min followed by a 4 °C hold step. Samples for the characterisation of senescence experiments were diluted to 12.5 ng/µl after reverse transcription. Due to low yield for some of the progeroid samples, 50 ng of cDNA for the trametinib versus control experiments was pre-amplified for 14 cycles. Pre-amplification followed the manufacturer’s instructions using TaqMan™ PreAmp Master Mix (4384266, Applied Biosystems™) and pooled TaqMan™ Gene Expression Assays (FAM) (4331182, TaqMan®). Cycling conditions were: 95 °C for 10 min, 14 cycles [of 95 °C for 15 s, 60 °C for 4 min], 99 °C for 10 min, followed by a 4 °C hold step. We used TaqMan™ Gene Expression Assay IDs: *AKAP17A* Hs00946624_m1, *ATM* Hs00175892_m1, *CASP3* Hs00234387_m1, *CASP7* Hs00169152_m1, *CHEK1* Hs00967506_m1, *GUSB* Hs00939627_m1, *HNRNPA0* Hs00246543_s1, *HNRNPA1* Hs01656228_s1, *HNRNPA2B1* Hs00242600_m1, *HNRNPD* Hs01086912_m1, *HNRNPH3* Hs01032113_g1, *HNRNPK* Hs00829140_s1, *HNRNPM* Hs00246018_m1, *HNRNPUL2* Hs00859848_m1, *IDH3B* Hs00199382_m1, *PNISR* Hs00369090_m1, *PPIA* Hs04194521_s1, *RB1* Hs01078066_m1, *SRSF1* Hs00199471_m1, *SRSF2* Hs00427515_g1, *SRSF3* Hs00751507_s1, *SRSF6* Hs00607200_g1, *SRSF7* Hs00196708_m1, *TP53* Hs01034249_m1, and *TRA2B* Hs00907493_m1.

### Real-time quantitative PCR (RT-qPCR)

RT-qPCR was performed using TaqMan™ Universal Mastermix II (4440048, Applied Biosystems™) and TaqMan™ Gene Expression Assays (FAM) (4331182, TaqMan®) in three biological and three technical replicates. The assay IDs are listed above in the pre-amplification section. RT-qPCR was performed on the Quantstudio 12 K platform (Applied Biosystems™) in 5 µl reactions using 384-well plates. 1 µl of diluted/pre-amplified cDNA was used per reaction alongside 0.25 µl of Taqman™ Gene Expression Assay (which corresponds to 900 nM primer and 250 nM probe). Cycling conditions were: 50 °C for 2 min, 95 °C for 10 min, followed by 40 cycles of 95 °C for 15 s and 60 °C for 1 min. Fluorescence intensity was captured at the end of each cycle. Ramp speeds were 1.6 °C per second for all transitions. Gene expression was calculated by the comparative C_T_ technique, relative to the geometric mean (untreated data) or mean (vehicle/trametinib treated data) expression level of three endogenous housekeeping genes (*GUSB*, *IDH3B* and *PPIA*), which had been empirically demonstrated to provide the most stable baseline for comparison within each dataset using the online RefFinder website [50, 51]. For assessment of gene expression, transcript levels in progeroid cells were normalised to the mean expression of each gene in wild-type control cells. For the trametinib treatment experiments, the data was normalised to the vehicle-treated controls.

### Statistics

The effect size or mean ± standard error of the mean (SEM) is reported in the text, with the full statistics reported in the tables. t test statistics for SAB and immunocytochemical staining were performed using Graphpad Prism version 9.4.1 for Windows (GraphPad Software, San Diego, California USA, www.graphpad.com). IBM SPSS Statistics for Windows version 27.0 programme (Released 2020; IBM Corp, Armonk, NY) was used to perform t tests for the RT-qPCR data. Graphs were produced using GraphPad Prism version 9.4.1. Error bars on the graphs represent the SEM unless otherwise stated.

## Results

### Senescence phenotypes in HGPS, WS and CS cells

**Fig. 1:**
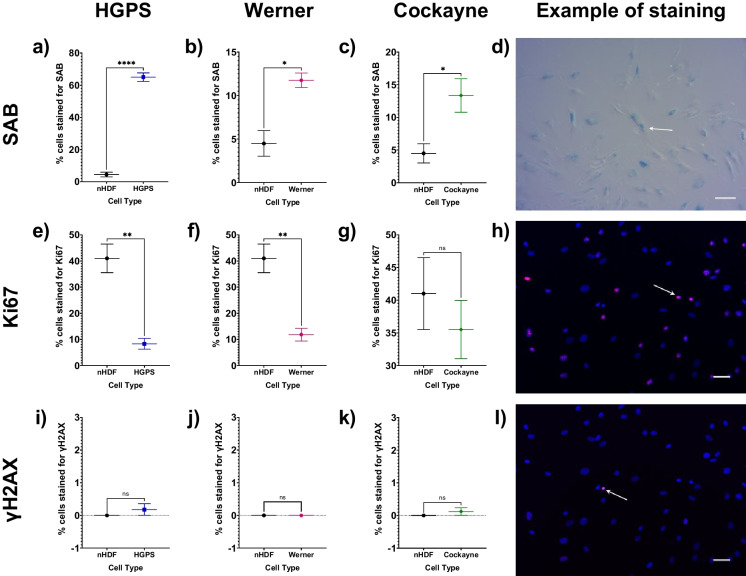
Senescence characteristics of progeroid cells compared to wild-type nHDFs. The mean ± standard error of the mean (SEM) are graphed. Asterisks denote a significant *p* value from a t test: * *p* < 0.05, ** *p* < 0.01, *** *p* < 0.001 and **** *p* < 0.0005. *n* = 3 for all experimental groups. Wild-type nHDFs are shown as black circles, HGPS cells: blue squares, Werner cells: pink hexagons, and Cockayne cells: green diamonds. **A**-**C**. Percentage of senescence-associated beta galactosidase (SAB)-positive cells for HGPS, Werner and Cockayne cells respectively compared to wild-type nHDFs. **D** Representative image of SAB staining (HGPS cells), 10 × magnification, scale bar denotes 100 µm, white arrow indicates a representative SAB stained-cell. **E**–**G** Percentage of cells stained for Ki67, a marker of proliferation, for HGPS, Werner and Cockayne cells respectively compared to wild-type nHDFs. **H** Representative image of Ki67 staining (Cockayne cells), 10 × magnification, scale bar denotes 50 µm, white arrow indicates a representative Ki67 stained-cell. **I**-**K** Percentage of cells stained for γH2AX, a marker of DNA damage repair, for HGPS, Werner and Cockayne cells respectively compared to wild-type nHDFs. **L** Representative image of γH2AX staining (Cockayne cells), 10 × magnification, scale bar denotes 50 µm, white arrow indicates a representative γH2AX stained-cell

**Fig. 2:**
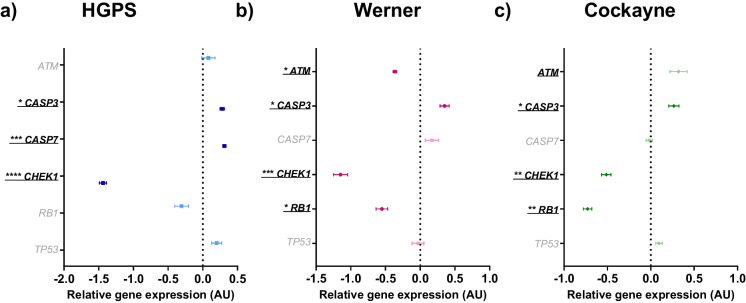
Forest plot of relative gene expression (arbitrary units, AU) of markers of apoptosis (*CASP3* and *CASP7*) and DNA damage repair (*ATM*, *CHEK1*, *RB1* and *TP53*) in progeroid cells compared against wild-type nHDFs. The mean ± standard error of the mean (SEM) are graphed. Asterisks denote a significant p value from a t test: * *p* < 0.05, ** *p* < 0.01, *** *p* < 0.001 and **** *p* < 0.0005. *n* = 3 for all experimental groups. Dark colouration of symbols indicates a significant *p* value, light colouration indicates non-significance. HGPS cells are shown as blue squares, Werner cells: pink hexagons, and Cockayne cells: green diamonds. **A** HGPS cells. **B** Werner cells. **C** Cockayne cells

Early passage fibroblasts from all three progeroid syndromes demonstrated higher levels of senescence than similar passage wild-type fibroblasts. SAB staining was 13.5-fold, 1.6-fold and twofold higher than nHDFs for HGPS, WS and CS, respectively (*p* < 0.0005, *p* = 0.0129 and *p* = 0.0401) as shown in Fig. 1A-C and Table 1. Cells from donors with HGPS and WS also demonstrated lower levels of proliferation (80% and 71% lower for HGPS and WS, respectively; *p* = 0.0050 and *p* = 0.0082; Fig. 1E and F), whereas cells from the phenotypically less severe CS patient demonstrated no differences in proliferation compared to controls (*p* = 0.048; Fig. 1G). Figure 1D, H and L show representative images of the staining methods used to measure senescence phenotypes (SAB, Ki67 and γH2AX staining respectively) with full staining data available in Table 1. We did not detect any differences in the number of γH2AX (DNA damage repair) foci in cells from progeroid donors compared with wild-type fibroblasts (Fig. 1I-K). The expression of several genes that control DNA damage repair were lower in the progeroid cells suggesting the progeroid cells have an impaired DNA damage response (Fig. 2A-C and Table 2). *CHEK1* expression was lower in all three types (HGPS: 144% lower, *p* < 0.0005, WS: 115% lower, *p* = 0.001, CS: 51% lower, *p* = 0.007). *RB1* expression was lower in WS and CS cells: 55% lower, *p* = 0.013, and 73% lower, *p* = 0.003, respectively. *ATM* expression was 37% lower in WS cells, *p* = 0.011. *TP53* expression was not significantly different between wild-type nHDFs and any progeroid cell type. Levels of apoptosis were higher in all three progeroid cell types (Fig. 2A-C and Table 2), with significantly higher levels of relative *CASP3* expression (HGPS: 28% higher, *p* = 0.025, WS: 35% higher, *p* = 0.023, CS: 27% higher, *p* = 0.046). Relative *CASP7* expression was 31% higher in HGPS cells (*p* < 0.0005), but was not significantly different in WS and CS cells (WS: 17% higher, *p* = 0.211. CS: 2% lower, *p* = 0.662).

**Table 1:** Wild-type nHDFs are compared against HGPS cells, Werner cells, and Cockayne cells for staining for senescence-associated beta galactosidase (SAB), Ki67 or γH2AX

Stain	Wild-type nHDFs		HGPS Untreated			Werner Untreated			Cockayne Untreated		
	Mean (%)	SEM	Mean (%)	SEM	*p* value	Mean (%)	SEM	*p* value	Mean (%)	SEM	*p* value
SAB	4.49	1.472	65.04	2.627	** < 0.0001**	11.75	0.8429	**0.0129**	13.35	2.563	**0.0401**
Ki67	41.01	5.473	8.29	2.055	**0.005**	11.83	2.419	**0.0082**	35.51	4.463	0.4796
γH2AX	0.00	0.00	0.18	0.179	0.3715	0.00	0.00	> 0.9999	0.12	0.1155	0.3574

The mean ± standard error of the mean (SEM) and t test *p* values are reported. Significant *p* values > 0.05 are emboldened. *n* = 3 for all experimental groups

**Table 2:** Wild-type nHDFs are compared against HGPS cells, Werner cells, and Cockayne cells for RT-qPCR analysis

Gene	Wild-type nHDFs		HGPS Untreated			Werner Untreated			Cockayne Untreated		
	Mean	SEM	Mean	SEM	*p* value	Mean	SEM	*p* value	Mean	SEM	*p* value
*AKAP17A*	0.0000	0.00280	0.1470	0.01363	**0.007**	-0.0546	0.11555	0.683	-0.1459	0.03168	**0.043**
*ATM*	0.0000	0.07705	0.0800	0.09539	0.550	-0.3651	0.02554	**0.011**	0.3204	0.09963	0.064
*CASP3*	0.0000	0.07229	0.2773	0.03158	**0.025**	0.3508	0.06528	**0.023**	0.2650	0.05853	**0.046**
*CASP7*	0.0000	0.01457	0.3100	0.02392	**0.000**	0.1697	0.09410	0.211	-0.0179	0.03508	0.662
*CHEK1*	0.0000	0.08541	-1.4388	0.05045	**0.000**	-1.1489	0.10135	**0.001**	-0.5133	0.05343	**0.007**
*HNRNPA0*	0.0000	0.06729	-0.2683	0.19968	0.272	-0.7537	0.26784	0.052	-0.0312	0.12356	0.835
*HNRNPA1*	0.0000	0.05322	-0.5208	0.01070	**0.001**	-0.4308	0.06006	**0.006**	-0.5611	0.03949	**0.001**
*HNRNPA2B1*	0.0000	0.07217	-0.2055	0.02496	0.055	-0.0397	0.07462	0.722	-0.9304	0.06115	**0.001**
*HNRNPD*	0.0000	0.05610	-0.1355	0.00805	0.075	0.2520	0.02337	**0.014**	-0.1257	0.11701	0.387
*HNRNPH3*	0.0000	0.04276	-0.0759	0.06390	0.379	0.1140	0.10229	0.362	-0.4689	0.03199	**0.001**
*HNRNPK*	0.0000	0.03673	-0.2728	0.08722	**0.045**	-0.1428	0.03650	0.051	-0.3678	0.02339	**0.001**
*HNRNPM*	0.0000	0.10042	-0.7215	0.10990	**0.008**	-0.9688	0.21861	**0.016**	-0.7633	0.10946	**0.007**
*HNRNPUL2*	0.0000	0.13141	0.1405	0.04493	0.369	-0.1551	0.15503	0.488	-0.2533	0.05284	0.148
*PNISR*	0.0000	0.08904	0.7377	0.11132	**0.007**	0.9260	0.06260	**0.001**	0.5193	0.05651	**0.008**
*RB1*	0.0000	0.09806	-0.3072	0.09847	0.092	-0.5524	0.08346	**0.013**	-0.7291	0.04874	**0.003**
*SRSF1*	0.0000	0.03662	-0.3201	0.05212	**0.007**	-0.2801	0.15955	0.217	-0.2279	0.02507	**0.007**
*SRSF2*	0.0000	0.16357	-1.2819	0.13558	**0.004**	-1.1881	0.33309	**0.033**	-0.3919	0.08658	0.102
*SRSF3*	0.0000	0.00971	-0.0848	0.06687	0.278	0.1734	0.02958	**0.005**	-0.0263	0.06912	0.741
*SRSF6*	0.0000	0.04396	0.0396	0.04482	0.563	0.3092	0.08235	**0.030**	0.0416	0.05593	0.590
*SRSF7*	0.0000	0.04921	-0.2368	0.02322	**0.012**	0.1172	0.01437	0.084	-0.2363	0.02035	**0.011**
*TP53*	0.0000	0.09066	0.1988	0.07199	0.161	-0.0329	0.08488	0.804	0.0943	0.03803	0.392
*TRA2B*	0.0000	0.04640	-0.0967	0.03050	0.157	0.0279	0.02613	0.628	0.1031	0.04419	0.183

The mean ± standard error of the mean (SEM) and t test *p* values are reported. Significant *p* values > 0.05 are emboldened. *n* = 3 for all experimental groups. All results are logged and normalised to the corresponding natural log of the wild-type nHDFs resulting in a mean of 0.0000 for all nHDFs

### Dysregulation of splicing factor expression in HGPS, WS and CS cells

**Fig. 3:**
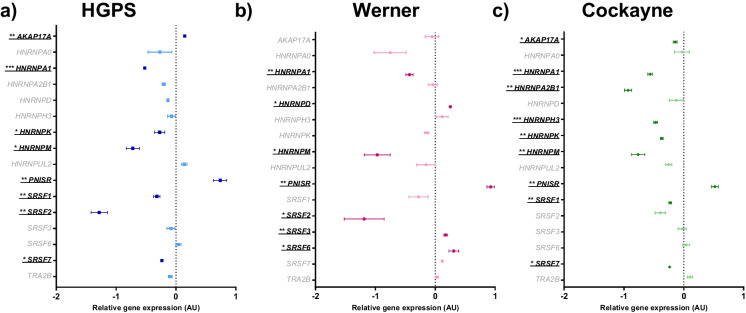
Forest plot of relative gene expression (arbitrary units, AU) of an a priori panel of splicing factors in progeroid cells compared against wild-type nHDFs. The mean ± standard error of the mean (SEM) are graphed. Asterisks denote a significant *p* value from a t test: * *p* < 0.05, ** *p* < 0.01, *** *p* < 0.001 and **** *p* < 0.0005. *n* = 3 for all experimental groups. Dark colouration of symbols indicates a significant *p* value, light colouration indicates non-significance. HGPS cells are shown as blue squares, Werner cells: pink hexagons, and Cockayne cells: green diamonds. **A** HGPS cells. **B** Werner cells. **C** Cockayne cells

Overall, most splicing factors were downregulated in progeroid syndromes compared to the wild type. 75% of the significant effects observed in HGPS were a decrease, with 43% and 89% of effects for WS and CS, respectively (Fig. 3A-C and Table 2). Some splicing factors demonstrated altered expression in one or more progeroid cell type; *HNRNPM*, *HNRNPA1* and *PNISR* expression were altered in all three cell types. *HNRNPM* expression was reduced by 72%, 97% and 76% for HGPS, WS and CS cells respectively (*p* = 0.008, 0.016 and 0.007). *HNRNPA1* expression was reduced by 52%, 43% and 56% lower for HGPS, WS and CS cells respectively (*p* = 0.001, 0.006 and 0.001) whilst *PNISR* expression was increased by 74%, 93% and 52% in HGPS, WS and CS cells respectively (*p* = 0.007, 0.001 and 0.008).

*SRSF2* expression was reduced by 128% and 119% in HGPS and WS cells respectively (*p* = 0.004 and 0.033), but not in CS cells. *SRSF1*, *SRSF7* and *HNRNPK* expression was reduced in HGPS and CS cells respectively, but not in WS cells; *SRSF1* = 32% and 23% (*p* = 0.007 and 0.007). *SRSF7* = 24% and 24% (*p* = 0.012 and 0.01) and *HNRNPK* = 27% and 37% (*p* = 0.045 and 0.001). *AKAP17A* was dysregulated in both HGPS and CS cells, although the directionality was different with slightly elevated expression in HGPS cells (15%; *p* = 0.007), but slightly reduced expression in CS cells (15%; *p* = 0.043). *HNRNPD*, *SRSF3* and *SRSF6* expression was altered in cells from the WS donor only with 25% (*p* = 0.014), 17% (*p* = 0.005) and 31% (*p* = 0.030) increases to expression respectively. Similarly, *HNRNPA2B1* and *HNRNPH3* expression was affected only in the CS donor, with levels reduced by 93% and 47% respectively (*p* = 0.001 and *p* = 0.001).

### Trametinib treatment reduced aspects of senescence in HGPS cells

**Fig. 4:**
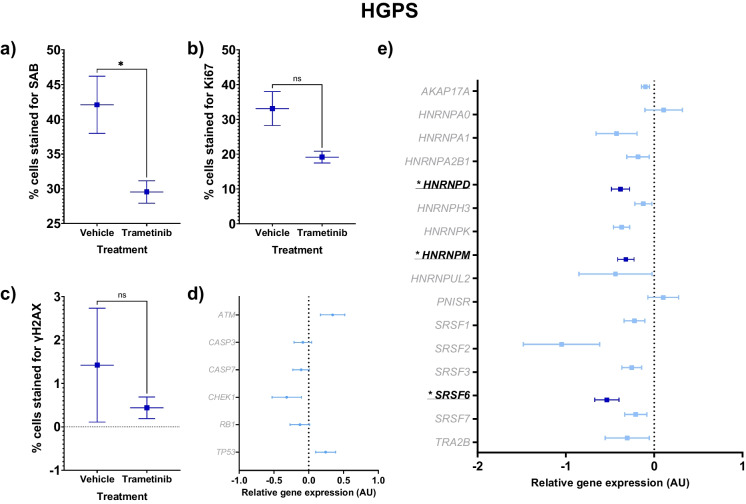
Senescence characteristics of HGPS cells (blue squares) treated with trametinib 10 µM compared to vehicle controls. The mean ± standard error of the mean (SEM) are graphed. Asterisks denote a significant *p* value from a t test: * *p* < 0.05, ** *p* < 0.01, *** *p* < 0.001 and **** *p* < 0.0005. *n* = 3 for all experimental groups. For gene expression graphs (D-E), a dark blue colouration denotes significance for a difference in trametinib-treated HGPS cells versus vehicle-treated HGPS cells, while non-significance is shown as light blue colouration. **A** Percentage of SAB positive cells. **B** Percentage of cells stained for Ki67, a marker of proliferation. **C** Percentage of cells stained for γH2AX, a marker of DNA damage repair. **D** Forest plot of relative gene expression (arbitrary units, AU) of *CASP3* and *CASP7*, markers of apoptosis, and *ATM*, *CHEK1*, *RB1* and *TP53*, markers of DNA damage repair. **E** Forest plot of relative gene expression (AU) of an a priori panel of splicing factors

HGPS cultures treated with the senomorphic drug, trametinib, demonstrated a lower senescent cell burden than vehicle-treated HGPS controls, and had decreased gene expression for three splicing factors (Tables 3 and 4 and Fig. 4A-E). SAB levels were 30% lower in the treated cells (*p* = 0.0471, Fig. 4A). Proliferation measured by Ki67 staining was 42% lower in the treated cells but was not significant (*p* = 0.0536, Fig. 4B). DNA damage repair (γH2AX staining and gene expression of *ATM*, *CHEK1*, *RB1* and *TP53*) and apoptosis (gene expression of *CASP3* and *CASP7*) did not show any significant changes with treatment (Fig. 4C-D). Trametinib treatment affected the gene expression of splicing factors involved with senescence (Fig. 4E). Gene expression was significantly decreased following treatment for *HNRNPD* (-38%; *p* = 0.027), *HNRNPM* (-32%; *p* = 0.048) and *SRSF6* (-54%; *p* = 0.042).

**Table 3:** Effects of trametinib treatment on staining for senescence-associated beta galactosidase (SAB), Ki67 or γH2AX in HGPS, Werner and Cockayne syndrome cells

Stain	DMSO treated HGPS		Trametinib treated HGPS			DMSO treated Werner		Trametinib treated Werner			DMSO treated Cockayne		Trametinib treated Cockayne		
	Mean (%)	SEM	Mean (%)	SEM	*p* value	Mean (%)	SEM	Mean (%)	SEM	*p* value	Mean (%)	SEM	Mean (%)	SEM	*p* value
SAB	42.09	4.1170	29.55	1.6170	**0.0471**	60.19	6.0040	61.00	7.3210	0.9359	4.94	0.4619	2.00	0.4503	**0.0104**
Ki67	33.10	4.8670	19.14	1.6970	0.0536	14.17	5.7450	2.32	0.4561	0.1089	41.39	3.5620	7.41	3.8340	**0.0029**
γH2AX	1.42	1.3110	0.44	0.2483	0.5033	0.27	0.2714	0.27	0.2714	> 0.9999	0.10	0.1039	0.07	0.0751	0.8265

Progeroid cell cultures are treated with a DMSO control or 10 µM trametinib. The mean ± standard error of the mean (SEM) and t test *p* values are reported. Significant *p* values > 0.05 are emboldened. *n* = 3 for all experimental groups

**Table 4:** Gene expression in HGPS cells treated with a DMSO control or 10 µM trametinib

Gene	DMSO treated HGPS		Trametinib treated HGPS		*p* value
	Mean	SEM	Mean	SEM	
*AKAP17A*	0.0000	0.07939	-0.0989	0.04495	0.339
*ATM*	0.0000	0.14735	0.3436	0.17585	0.209
*CASP3*	0.0000	0.09790	-0.0846	0.12557	0.623
*CASP7*	0.0000	0.06298	-0.1093	0.11819	0.460
*CHEK1*	0.0000	0.10604	-0.3162	0.21142	0.252
*HNRNPA0*	0.0000	0.03831	0.1074	0.21222	0.665
*HNRNPA1*	0.0000	0.04375	-0.4259	0.23231	0.205
*HNRNPA2B1*	0.0000	0.06580	-0.1838	0.12724	0.269
*HNRNPD*	0.0000	0.04324	-0.3813	0.10334	**0.027**
*HNRNPH3*	0.0000	0.12678	-0.1232	0.09660	0.483
*HNRNPK*	0.0000	0.19698	-0.3700	0.09144	0.164
*HNRNPM*	0.0000	0.06595	-0.3206	0.09250	**0.048**
*HNRNPUL2*	0.0000	0.02529	-0.4382	0.41338	0.400
*PNISR*	0.0000	0.05944	0.1025	0.17489	0.609
*RB1*	0.0000	0.17780	-0.1268	0.14084	0.606
*SRSF1*	0.0000	0.02077	-0.2233	0.11684	0.193
*SRSF2*	0.0000	0.15928	-1.0493	0.43229	0.085
*SRSF3*	0.0000	0.14506	-0.2533	0.11203	0.239
*SRSF6*	0.0000	0.11818	-0.5360	0.13775	**0.042**
*SRSF7*	0.0000	0.01591	-0.2092	0.12513	0.235
*TP53*	0.0000	0.04728	0.2429	0.14423	0.185
*TRA2B*	0.0000	0.03686	-0.3055	0.25036	0.294

Vehicle-treated cells are compared against treated cells for RT-qPCR analysis. The mean ± standard error of the mean (SEM) and t test *p* values are reported. Significant *p* values > 0.05 are emboldened. *n* = 3 for all experimental groups. All results are logged and normalised to the DMSO treated control resulting in a mean of 0.0000 for these cells

### Trametinib had no effect on senescence phenotypes in Werner syndrome cells

**Fig. 5:**
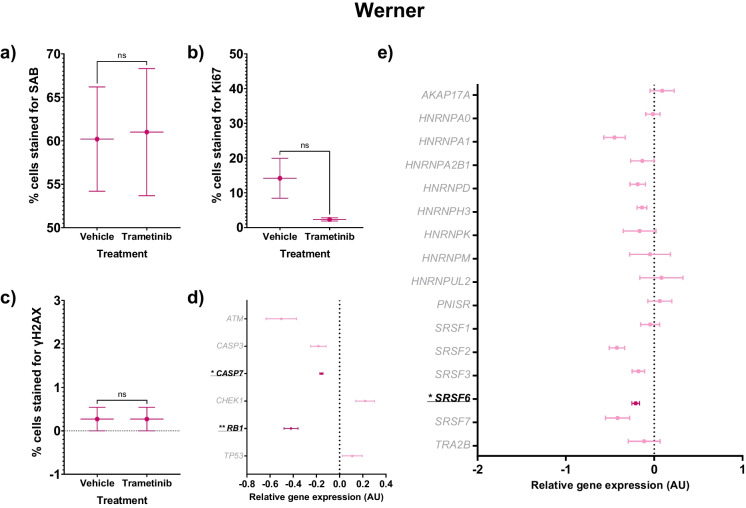
Senescence characteristics of Werner cells (pink hexagons) treated with trametinib 10 µM compared to vehicle controls. The mean ± standard error of the mean (SEM) are graphed. Asterisks denote a significant *p* value from a t test: * *p* < 0.05, ** *p* < 0.01, *** *p* < 0.001 and **** *p* < 0.0005. *n* = 3 for all experimental groups. For gene expression graphs (**D**-**E**), a dark pink colouration denotes significance for a difference in trametinib-treated Werner cells versus vehicle-treated Werner cells, while non-significance is shown as light pink colouration. **A** Percentage of SAB positive cells. **B** Percentage of cells stained for Ki67, a marker of proliferation. **C** Percentage of cells stained for γH2AX, a marker of DNA damage repair. **D** Forest plot of relative gene expression (arbitrary units, AU) of *CASP3* and *CASP7*, markers of apoptosis, and *ATM*, *CHEK1*, *RB1* and *TP53*, markers of DNA damage repair. **E** Forest plot of relative gene expression (AU) of an a priori panel of splicing factors

Werner cells did not show any changes in senescence, proliferation or DNA damage repair in response to trametinib treatment, however gene expression of *CASP7* and *SRSF6* were decreased (Tables 3 and 5 and Fig. 5A-E). Levels of senescence (SAB staining) did not show any change in response to the treatment (Fig. 5A). Werner cells had a similar response to HPGS in terms of proliferation (an 83% decrease), but this was not significant (*p* = 0.1089), and γH2AX staining was unchanged (Fig. 5B-C). *RB1* gene expression was decreased by 42% with treatment (*p* = 0.004), but *ATM*, *CHEK1* and *TP53* expression remained unchanged (Fig. 5D). Apoptosis was affected by the treatment (Fig. 5D): *CASP3* gene expression was not changed, but *CASP7* expression was significantly lower (-16%; *p* = 0.043). *SRSF6* expression was lower with trametinib treatment (-21%; *p* = 0.037, Fig. 5E).

**Table 5:** Gene expression in Werner cells treated with a DMSO control or 10 µM trametinib

Gene	DMSO treated Werner		Trametinib treated Werner		*p* value
	Mean	SEM	Mean	SEM	
*AKAP17A*	0.0000	0.07775	0.0899	0.13752	0.600
*ATM*	0.0000	0.23276	-0.5034	0.13155	0.133
*CASP3*	0.0000	0.18080	-0.1829	0.06654	0.396
*CASP7*	0.0000	0.05171	-0.1568	0.01412	**0.043**
*CHEK1*	0.0000	0.43087	0.2209	0.08056	0.641
*HNRNPA0*	0.0000	0.17741	-0.0153	0.08047	0.941
*HNRNPA1*	0.0000	0.20095	-0.4500	0.12193	0.128
*HNRNPA2B1*	0.0000	0.08419	-0.1348	0.13226	0.439
*HNRNPD*	0.0000	0.18667	-0.1868	0.08756	0.416
*HNRNPH3*	0.0000	0.02166	-0.1390	0.05445	0.077
*HNRNPK*	0.0000	0.24257	-0.1654	0.18525	0.617
*HNRNPM*	0.0000	0.37251	-0.0475	0.23086	0.919
*HNRNPUL2*	0.0000	0.56743	0.0817	0.24466	0.901
*PNISR*	0.0000	0.07420	0.0635	0.13543	0.702
*RB1*	0.0000	0.04035	-0.4196	0.06019	**0.004**
*SRSF1*	0.0000	0.01980	-0.0463	0.10754	0.694
*SRSF2*	0.0000	0.57286	-0.4222	0.08741	0.507
*SRSF3*	0.0000	0.09257	-0.1789	0.07099	0.200
*SRSF6*	0.0000	0.05241	-0.2093	0.04376	**0.037**
*SRSF7*	0.0000	0.12667	-0.4149	0.13621	0.090
*TP53*	0.0000	0.14398	0.1088	0.08559	0.551
*TRA2B*	0.0000	0.02057	-0.1139	0.18060	0.593

Vehicle-treated cells are compared against treated cells for RT-qPCR analysis. The mean ± standard error of the mean (SEM) and t test *p* values are reported. Significant *p* values > 0.05 are emboldened.* n* = 3 for all experimental groups. All results are logged and normalised to the DMSO treated control resulting in a mean of 0.0000 for these cells

### Trametinib treatment reduced aspects of senescence in Cockayne syndrome cells

**Fig. 6:**
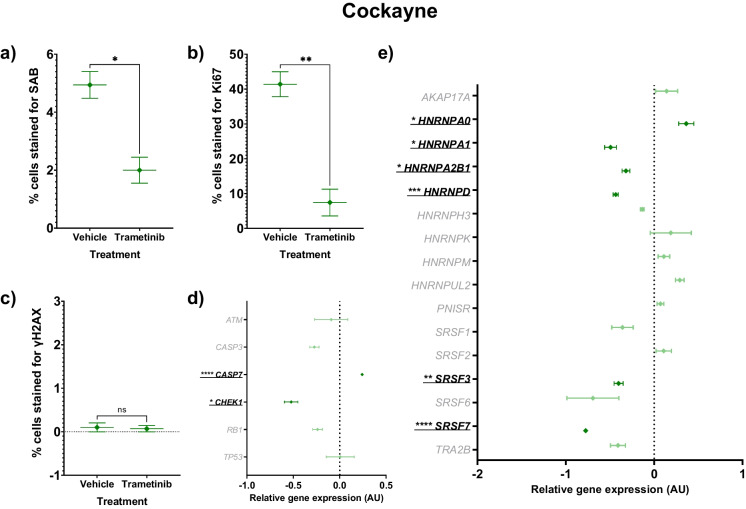
Senescence characteristics of Cockayne cells (green diamonds) treated with trametinib 10 µM compared to vehicle controls. The mean ± standard error of the mean (SEM) are graphed. Asterisks denote a significant *p* value from a t test: * *p* < 0.05, ** *p* < 0.01, *** *p* < 0.001 and **** *p* < 0.0005. *n* = 3 for all experimental groups. For gene expression graphs (**D**-**E**), a dark green colouration denotes significance for a difference in trametinib-treated Cockayne cells versus vehicle-treated Cockayne cells, while non-significance is shown as light green colouration. **A** Percentage of SAB positive cells. **B** Percentage of cells stained for Ki67, a marker of proliferation. **C** Percentage of cells stained for γH2AX, a marker of DNA damage repair. **D** Forest plot of relative gene expression (arbitrary units, AU) of *CASP3* and *CASP7*, markers of apoptosis, and *ATM*, *CHEK1*, *RB1* and *TP53*, markers of DNA damage repair. **E** Forest plot of relative gene expression (AU) of an a priori panel of splicing factors

Senescence and proliferation are decreased with trametinib treatment in Cockayne cells and six splicing factors have altered gene expression (Tables 3 and 6 and Fig. 6A-E). SAB staining was 60% lower (*p* = 0.0104) in treated cells compared to vehicle-treated CS controls (Fig. 6A). Proliferation was significantly lower in treated Cockayne cells with an 82% reduction in Ki67 staining (*p* = 0.0029, Fig. 6B). γH2AX staining levels remained low and were unchanged with treatment (Fig. 6C). *CHEK1* gene expression was decreased by 52% in trametinib-treated cells (*p* = 0.020), but the gene expression of other markers of DNA damage repair, *ATM*, *CHEK1* and *TP53*, were unchanged (Fig. 6D). *CASP3* expression was unchanged, but *CASP7* expression was increased by 24% with treatment (*p* < 0.0005, Fig. 6D). Trametinib treatment had effects on more splicing factors’ gene expression in Cockayne cells compared to HGPS and Werner cells. *HNRNPA0* expression levels were higher following treatment (36%; *p* = 0.030), whilst *HNRNPA1*, *HNRNPA2B1, HNRNPD*, *SRSF3* and *SRSF7* expression was lower (49%; *p* = 0.029; 32%; *p* = 0.030; 44%; *p* < 0.0005; 40%; *p* = 0.004 and 77%; *p* < 0.0005 respectively, Fig. 6E).

**Table 6:** Gene expression in Cockayne cells treated with a DMSO control or 10 µM trametinib

Gene	DMSO treated Cockayne		Trametinib treated Cockayne		*p* value
	Mean	SEM	Mean	SEM	
*AKAP17A*	0.0000	0.07884	0.1410	0.12482	0.394
*ATM*	0.0000	0.18303	-0.0916	0.17808	0.738
*CASP3*	0.0000	0.08686	-0.2734	0.04976	0.052
*CASP7*	0.0000	0.00541	0.2417	0.01002	**0.000**
*CHEK1*	0.0000	0.11952	-0.5229	0.07282	**0.020**
*HNRNPA0*	0.0000	0.07020	0.3633	0.08528	**0.030**
*HNRNPA1*	0.0000	0.13207	-0.4939	0.06731	**0.029**
*HNRNPA2B1*	0.0000	0.08542	-0.3190	0.04467	**0.030**
*HNRNPD*	0.0000	0.02346	-0.4350	0.02832	**0.000**
*HNRNPH3*	0.0000	0.06340	-0.1357	0.02037	0.111
*HNRNPK*	0.0000	0.09832	0.1883	0.23219	0.497
*HNRNPM*	0.0000	0.09515	0.1104	0.06560	0.394
*HNRNPUL2*	0.0000	0.45255	0.2901	0.04871	0.588
*PNISR*	0.0000	0.08017	0.0710	0.03888	0.470
*RB1*	0.0000	0.08707	-0.2386	0.05264	0.079
*SRSF1*	0.0000	0.06496	-0.3585	0.12077	0.059
*SRSF2*	0.0000	0.47592	0.1084	0.08712	0.834
*SRSF3*	0.0000	0.04399	-0.4022	0.05133	**0.004**
*SRSF6*	0.0000	0.10491	-0.6934	0.29393	0.090
*SRSF7*	0.0000	0.00882	-0.7748	0.01598	**0.000**
*TP53*	0.0000	0.06693	0.0049	0.15123	0.978
*TRA2B*	0.0000	0.12949	-0.4098	0.08471	0.057

Vehicle-treated cells are compared against treated cells for RT-qPCR analysis. The mean ± standard error of the mean (SEM) and t test *p* values are reported. Significant *p* values > 0.05 are emboldened. *n* = 3 for all experimental groups. All results are logged and normalised to the DMSO treated control resulting in a mean of 0.0000 for these cells

## Discussion

Progeroid syndromes are life-limiting rare diseases that share many similarities with the processes that are involved in normal ageing and age-related diseases. Senotherapeutic compounds can be used to target ageing, age-related disease and premature ageing. With few treatments available for progeroid syndromes, finding new senotherapeutics is important. Some senotherapeutic compounds like trametinib are thought to work by restoring levels of the splicing regulatory factors that control alternative splicing decisions. We first investigated if splicing factor expression profiles were altered in dermal fibroblasts from donors with Hutchinson-Gilford Progeria syndrome, Werner syndrome and Cockayne syndrome, and secondly, if a known senomorphic drug, trametinib, could impact senescence kinetics and splicing factor expression in these cell populations. We observed that cultures of early passage primary dermal fibroblast cells from individuals with some progeroid syndromes demonstrated elevated senescent cell load and altered splicing factor expression compared to that observed in early passage wild-type cells. Furthermore, we saw that trametinib was able to influence the expression levels of some splicing factors and influence some, but not all, aspects of the senescent cell phenotype in these cells.

We noted elevated senescent cell load in the more severe syndromes (HGPS and WS) compared with cells from the more moderately affected CS patient, and disease severity and senescent cell load also correlated with degree of ‘rescue’ upon trametinib treatment. WS and HGPS cells demonstrated fewer changes to splicing factor expression and a more moderate effect on senescent cell load; upon treatment with trametinib CS cells demonstrated a 71% decrease in senescent cell load (though this effect may be exaggerated as the percentage of SAB-stained cells in the vehicle treatment was low) compared with 33% for HGPS and no change for WS (Table 3, Figs. 4A, E, 5A, E, 6A and E). This may suggest that the most severe progerias may have an increased prevalence of terminally and irreversibly senescent cells.

In line with previous studies on senescence induced in wild type cells by replicative senescence, we also observed changes to the portfolio of splicing factors expressed. Some of these splicing regulatory factors have previously been associated with ageing phenotypes in humans. *HNRNPA1* and *HNRNPA2B1* have previously been reported to be associated with parental lifespan in human populations [25] and with senescence in human primary cells [24, 52], whereas *HNRNPM* and *AKAP17A* have previously been reported to demonstrate predictive associations with cognitive decline and loss of muscle strength [53, 54]. Although we did not directly address alternative splicing patterns in our progeroid cultures, previous work in a mouse model of HGPS has documented that whilst the number of alternatively spliced genes is similar to that observed in wild-type mice in young animals, the number of alternatively spliced genes in the HPGS model mice was altered relative to those seen in wild-type mice as the animals age [55]. It is noteworthy that in the two cell lines (HGPS and CS) where we were able to demonstrate attenuation of some senescence phenotypes following trametinib treatment; three and six splicing factors were altered in response to treatment, compared with the situation for WS, where we detected changes in expression for a single splicing factor only.

Another interesting and surprising observation was the overall low level of γH2AX staining in our progeroid cell cultures. This is counterintuitive given that these are all syndromes of DNA damage, and elevated damage has previously been reported to be elevated in progeroid syndromes [56–59]. However, it is important to note that γH2AX is more specifically a marker of the initiation of the DNA damage repair response [60, 61]. DNA damage repair can be inhibited in progeroid cells and other studies have reported that the intensity of γH2AX foci was low in HGPS cells [3, 62]. The relative lack of γH2AX foci we observe in our progeroid cell cultures may therefore reflect the low proportion of repair-competent cells in these cultures, rather than low levels of DNA damage per se. The expression of genes that encode markers of DNA damage repair supports this; *CHEK1*, *ATM* and *RB1* expression were significantly decreased in the progeroid cell types. *CHEK1* integrates signals from *ATM* and *ATR* [63], affected all three progeroid cell types, and was the most affected gene of the four DNA damage repair genes studied. Our data suggests that repair is impacted in the progeroid cells compared with wild-type nHDFs. With trametinib treatment, only *RB1* in WS cells and *CHEK1* in CS cells were affected. Given that trametinib inhibits MEK which interacts with the pathways that govern these genes, it is likely that these two effects are a direct result of the gene regulatory network rather than an indication of trametinib markedly changing DNA damage repair response. A marker of the execution phase of apoptosis (*CASP3*) is elevated in all three untreated progeroid cell lines, which may provide evidence for the presence of elevated damage in these cells relative to wild-type controls.

Trametinib as a drug is often used for cancer chemotherapy in combination [64]. The reduction in Ki67 is therefore unsurprising when we consider that trametinib is a known anti-neoplastic therapy [45]. Trametinib commonly has a variety of side effects including gastrointestinal issues, but this is during a high dose daily treatment programme. Single doses have been enough in a senotherapeutic context to give benefit so it may be that future therapies using trametinib for age-related diseases would use a single dose model. Senescent cells can show a level of heterogeneity and there is no single definitive marker [65, 66]. Senescence can be induced by several means: replication (via telomere attrition), stress (such as the accumulation of mutations due to poor nuclear stability in HGPS) and/or oncogenes [67–69]. However, there exists a lack of clarity over whether senescence biomarkers exist that are associated with specific subtypes of senescence. Several studies suggest that senomorphic compounds may target only SASP-induced (paracrine) senescence (a form of stress-induced senescence) [70, 71]. It is possible therefore, that treatments such as trametinib may target only subsets of senescent cells, so that conditions where the balance of subtypes is disturbed may show differential effects on rescue. For example, cells in which senescence has arisen because of catastrophic DNA damage may act differently to those which have arisen because of SASP-induced paracrine senescence. In the former there will be an ongoing signal for senescence, whilst in the latter, once the inflammatory milieu has been normalised, senescence may be more reversible. Our results from this study are consistent with this hypothesis. An interesting question would be whether we see similar effects in later passage cells from donors with progeroid syndromes. Such cells are, in effect, prematurely senescent, and share features with wild-type cells at later passages. Whilst the progeroid cells at an early passage may be ‘chronologically’ young, they appear ‘biologically’ aged. In the current work, we have carried out our experiments on progeroid cells at relatively early passage, because the cultures become senescent much earlier than wild-type cells. The effects one might observe using later passage cells are difficult to predict, but the increase in transcriptional noise and stochastic variation that occurs during even normal cellular ageing [72], may be amplified in these cells, meaning that consistent effects on gene expression and cellular phenotypes may be harder to detect.

Many senomorphic compounds cause biphasic dose responses in cells. Trametinib has been observed to exert different senotherapeutic effects at the 1—10 µM range compared to 20 µM doses in cells [28]. A 10 µM dose was chosen for this study based on this research, but it is possible that with a lower dose in the range the cells may show more restoration of splicing factor expression. A 20 µM dose of trametinib may have no effect on senescence, and, as our results suggest, a 10 µM dose may be sufficient to restore the responsiveness of splicing factor expression, but a 1 µM dose may be the dosage that can produce the best response. This may be as a result of a hormetic effect. A hormetic effect is when a cell responds to a minor stressor and overcompensates to the point that the stressor causes a slight benefit to the cell overall [73]. Several compounds such as resveratrol and metformin show this type of hormetic effect [74, 75]. Although trametinib is more specific in the mechanism that it targets compared to resveratrol or metformin, this type of dose effect is still common in compounds that target tightly controlled and highly autoregulated pathways, such as the networks that control splicing factor expression and cell fate. Autoregulation and cross-regulation have been noted in the MEK/ERK pathway that trametinib targets [32].

One caveat of this work is that the cells studied were dermal fibroblasts, so other cell types from a person with a progeroid syndrome may have different characteristics and responses to trametinib. Other subtypes of Cockayne syndrome, or other progeroid syndromes, such as Bloom syndrome and Xeroderma Pigmentosum, may not be as rescuable as the syndromes examined here. Further research is needed to uncover if these findings are replicated in other progeroid syndromes. Another caveat is that, although progeroid syndromes are widely considered a reasonable model for ‘normal’ ageing, given that progerias are syndromic in nature, they may not necessarily reflect what happens in normal ageing when used as a model [76]. It is interesting that trametinib is capable of partially rescuing the phenotype of these cells when the phenotypes are so severe. In another study, using a mouse model of HGPS, the senomorphic drug resveratrol was able to alleviate some features of the premature ageing phenotype [31]. This gives more evidence in support of the idea that senomorphic and senotherapeutic drugs may help in the progeroid syndromes [39, 76].

Our results are consistent with other studies that show that cells from individuals with progeroid syndromes display characteristics consistent with accelerated cellular and molecular ageing, which may be amenable to future therapeutic targeting [76]. Several senomorphic drugs, such as trametinib and resveratrol show rescue of senescent cell populations in contradiction to Terzi et al*.*’s definition of an “irreversible state of cell cycle arrest” [26, 28, 77]. Our body of work suggests that the most senescent and most DNA damaged cells are irreversibly senescent, but senomorphic drugs can reverse senescence in cells up to a certain point, giving more weight to the theory of stages of senescence, e.g. a reversible pre-senescent stage [68, 78].

Senotherapeutic and senomorphic drugs represent an intriguing way to think about treating progeroid disease as well as age-related disease. As our population ages, we have an increased burden of age-related disease, which means that more therapies will be needed to sustain a healthy population [79]. If compounds are able to address the underpinning mechanisms behind several different age-related diseases, then this could have more impact than attempting to treat each age-related disease individually. Our findings suggest that trametinib and other senotherapeutic compounds could be examined as an additional therapeutic angle for people with progeroid syndromes, and further evidences the notion that mRNA splicing factor dysregulation is a key cellular hallmark of ageing.
